# Associations of emotional/behavioral problems with accelerometer-measured sedentary behavior, physical activity and step counts in children with autism spectrum disorder

**DOI:** 10.3389/fpubh.2022.981128

**Published:** 2022-10-10

**Authors:** Hailin Li, Bijun Shi, Xin Wang, Muqing Cao, Jiajie Chen, Siyu Liu, Xiaoling Zhan, Chengkai Jin, Zhaohuan Gui, Jin Jing, Yanna Zhu

**Affiliations:** ^1^Department of Maternal and Child Health, School of Public Health, Sun Yat-sen University, Guangzhou, China; ^2^Department of Pediatrics, Third Affiliated Hospital of Guangzhou Medical University, Guangzhou, China

**Keywords:** emotional/behavioral problems, accelerometer-measured, sedentary behavior, physical activity, step counts, autism spectrum disorder

## Abstract

**Background:**

The evidence for associations of emotional/behavioral status with sedentary behavior (SB), physical activity (PA) and step counts is scarce in children with autism spectrum disorder (ASD). Also, ASD-related deficiencies may affect actual levels of PA. We aimed to describe accelerometer-measured SB, PA and step counts in children with ASD, and to examine the associations of emotional/behavioral problems with SB, PA and step counts after assessing associations between accelerometer-measured SB, PA and step counts and ASD-related deficiencies.

**Methods:**

A total of 93 ASD children, aged 6–9 years, were recruited from the Center for Child and Adolescent Psychology and Behavioral Development of Sun Yat-sen University in Guangzhou, China. Participants wore an accelerometer for seven consecutive days. Of the original 93, 78 participants' accelerometer-measured valid PA were obtained, and the data were shown as time spent in SB, light, moderate, moderate-to-vigorous and vigorous PA, and step counts. Participants' emotional/behavioral problems were assessed via the Strengths and Difficulties Questionnaire (SDQ), and anxiety symptoms were evaluated by the Screen for Child Anxiety Related Emotional Disorders (SCARED). ASD-associated deficiencies include restricted repetitive behaviors (Repetitive Behavior Scale-Revised), poor social competence (Social Responsiveness Scale Second Edition) and motor development restrictions (Developmental Coordination Disorder Questionnaire).

**Results:**

Of the 78 participants, daily vigorous PA (VPA) and moderate-to-vigorous PA (MVPA) averaged 15.62 and 51.95 min, respectively. After adjustment for covariates, SDQ emotional symptoms (β = −0.060, *p* = 0.028) were inversely associated with the average daily minutes in VPA. Meanwhile, SDQ emotional symptoms (β = −0.033, *p* = 0.016) were inversely associated with the average daily MVPA minutes in the crude model. After adjustment for covariates, SCARED somatic/panic (β = −0.007, *p* = 0.040) and generalized anxiety (β = −0.025, *p* = 0.014) were negatively associated with the average daily VPA minutes; SCARED total anxiety (β = −0.006, *p* = 0.029) was conversely associated with daily MVPA duration. After adjustment for covariates, no significant associations between accelerometer-measured SB, PA and step counts and ASD-related deficiencies were found (*p* > 0.05).

**Conclusions:**

Accelerometer-measured SB, PA and step counts showed no associations with ASD-related deficiencies. On this basis, we further found that the emotional symptoms were inversely associated with VPA and MVPA. These results emphasize the importance of VPA and MVPA in children with ASD. The longitudinally investigations on the directionality of these associations between emotional symptoms with VPA and MVPA are needed in the future.

## Highlights

- We described accelerometer-measured SB, PA, and step counts in children with ASD.
- We assessed associations between accelerometer-measured SB, PA and step counts and ASD-associated deficiencies (restrictive repetitive behaviors, poor social competence and motor development restrictions) in children with ASD.
- We examined associations of emotional/behavioral problems with accelerometer-measured SB, PA and step counts in children with ASD.

## Introduction

A high rate of children with autism spectrum disorders (ASD), a neuropsychiatric disorder with restricted repetitive behaviors (RRBs) such as repetitive body movements, sensory-stimulant behaviors, object-manipulation behaviors, restrictive interests, insistence on sameness, and repetitive language, as well as with poor social interaction, experience concomitant emotional and behavioral problems (1–3). In fact, the co-existing emotional disturbance and maladaptive behaviors like anxiety, tantrums, sabotage, aggression, hyperactivity and self-injurious are frequent in ASD regardless of age, ability, or schooling (4). The number of problems with emotion regulation and behavior are closely linked to the severity of social deficits as well as to RRBs (5, 6). Additionally, these problems increase the risk of comorbidities for a range of psychiatric disorders including depression, anxiety disorder, behavioral disorder, attention-deficit/hyperactivity disorder, and oppositional defiant disorder (6, 7).

The occurrence of emotional/behavioral problems interferes with rehabilitation training, the quality of life, contributes to substantial impairments in social competence, to academic and occupational underachievement and poor clinical outcomes (8, 9). Clinically, irritability and anxiety are often the reasons families seek unconventional therapies, medical interventions and hospitalization for their ASD children (10). The expenses incurred on the medical treatment and rehabilitation training for ASD will undoubtedly bring heavy economic burden on families and society. This shows that the elimination or attenuation of the concomitant emotional/behavioral problems can be equal or greater concern than control of core features of ASD (11).

Currently, studies have demonstrated the limited improvement of psychotropic medication and behavioral interventions on mental health outcomes in children with ASD (12, 13). Given the benefits of physical activity (PA) among typical children, PA as an appropriate strategy for decreasing ASD outcomes worthy of exploring and studying (14). Previous studies have demonstrated that PA-based interventions can effectively ameliorate autism degree, core symptoms of RRBs and social dysfunction (15–17). Also, ASD children who engage in physical exercise demonstrated substantial improvements in adaptive living skills, communication ability, executive functioning, motor skills, sensory processing skills, and academic engagement (14, 18). In general, PA appears to have benefits for children with ASD, a more rigorous and objective design to determine what intensity and duration of PA can be associated with improved mental health is clearly needed. Furthermore, sedentary behavior (SB) may contribute to adverse health outcomes in children independent of PA (19). To date, there is a relative paucity of studies that have investigated the associations of emotional/behavioral problems with accelerometer-measured SB, PA and step counts in children with ASD. To overcome the challenges of sedentary and inactive lifestyles and an emotional/behavioral problem in ASD children (2, 3, 20), a first step may be to set up whether cross-sectional relations exist. Most of previous studies collected self- and parent-reported information about SB and PA, which can be subject to report and recall bias (21). Moreover, ASD-related deficiencies including RRBs, poor social competence and motor development restrictions may interfere with PA participation levels (20). It is beneficial to assess the associations between participants' PA engagement and ASD-related deficiencies in order to account for these potential confounding factors on PA levels. Only a handful of studies have considered ASD-related defects that impact levels of SB, PA and step counts, and until now the results were not clear cut (18, 22). Accordingly, the present study had two main aims: (a) describe the accelerometer-measured levels of SB, PA and step counts of children with ASD; (b) examine associations of emotional/behavioral problems with accelerometer-measured SB, PA and step counts on the basis of assessing associations between SB, PA and step counts and ASD-associated deficiencies (RRBs, poor social competence and motor development restrictions) in children with ASD.

## Materials and methods

### Study design and participants

A cross-sectional study was performed from July 2018 to May 2019. A total of 93 ASD children, aged 6–9 years, were recruited from the Center for Child and Adolescent Psychology and Behavioral Development of Sun Yat-sen University in Guangzhou, China, excluding those who were taking medications for the core symptoms and psychological comorbidities of autism, had diseases affecting motor function, unable to use the accelerometer, and undergoing exercise intervention. Participants who received intervention trainings including the behavioral interventions, educational interventions, social interventions, and the integrated intervention model were not excluded. Of the original 93, 15 were removed due to invalid accelerometer data with a final sample of 78 ASD children, and there was no substantial difference in the distribution of overall demographic information between enrolled and excluded study participants.

The children obtained a diagnosis of autism, and identified as meeting the Diagnostic and Statistical Manual of Mental Disorders, 5th Edition (DSM-5) criteria. Childhood Autism Rating Scale (CARS) was conducted to ascertain the diagnosis and to distinguish between mild-to-moderate and severe ASD by two experienced developmental and behavioral pediatricians. According to the manual, the CARS score between 30 and 36.5 is indicative of mild-to-moderate ASD, and the score between 37 and 60 stands for severe ASD (23). We applied the full-scale intelligence quotient (FSIQ) derived from Wechsler Intelligence Scale for Children 4th Edition (WISC-IV) (24) to depict ASD children's general intellectual functioning and then split them into two groups: FSIQ < 70 and FSIQ ≥ 70 (25). This study was conducted in accordance with Good Clinical Practice guidelines, Declaration of Helsinki principles, and the Ethical Committee of the School of Public Health, Sun Yat-Sen University (No. 023 [2018]).

### Accelerometer measured SB and PA

The Actigraph GT3X+ accelerometer (Actigraph LLC, Pensacola, FL, USA) was used to measure SB, PA and step counts (26, 27). This is a compact (3.8 × 3.7 × 1.8 cm), lightweight (27 g) monitor from the new generation of Actigraph devices. Participants and their parents were instructed to wear an accelerometer on the right hip for seven consecutive days (5 working days and 2 weekend days), except while sleeping, bathing and swimming, and parents have been advised to supervise their children's accelerometer use. Accelerometer data were originally collected at 30 Hz and then integrated to 15-second epochs using the normal-frequency filter within ActiLife version 6 software (ActiGraph, Pensacola, FL, USA). Accelerometer files were screened for wear time via Troiano algorithm (28); valid wear was defined as ≥4 days (3 work days plus 1 weekend day) with ≥10 h per day. Non-wear periods were specified as ≥20 consecutive minutes of zero counts per minute (CPM). PA volume were expressed as CPM, and Evenson cutoff points to define PA intensities included: SB = 0–100 CPM; light PA (LPA) = 101–2,295 CPM; moderate PA (MPA) = 2296–4011 CPM; and vigorous PA (VPA) ≥4,012 CPM (29). Accelerometer output signals were also recorded as steps per minute and these have been validated (30). The accelerometer data were converted into time (hours/minutes) per valid day in SB, LPA, MPA, VPA and moderate-to-vigorous PA (MVPA), and step counts per valid day and minute. ASD children were categorized as meeting the PA guidelines (PAG) of ≥ 60 min/d MVPA according to the World Health Organization 2020 guidelines on PA and SB (31).

### Procedures

**Figure 1:**
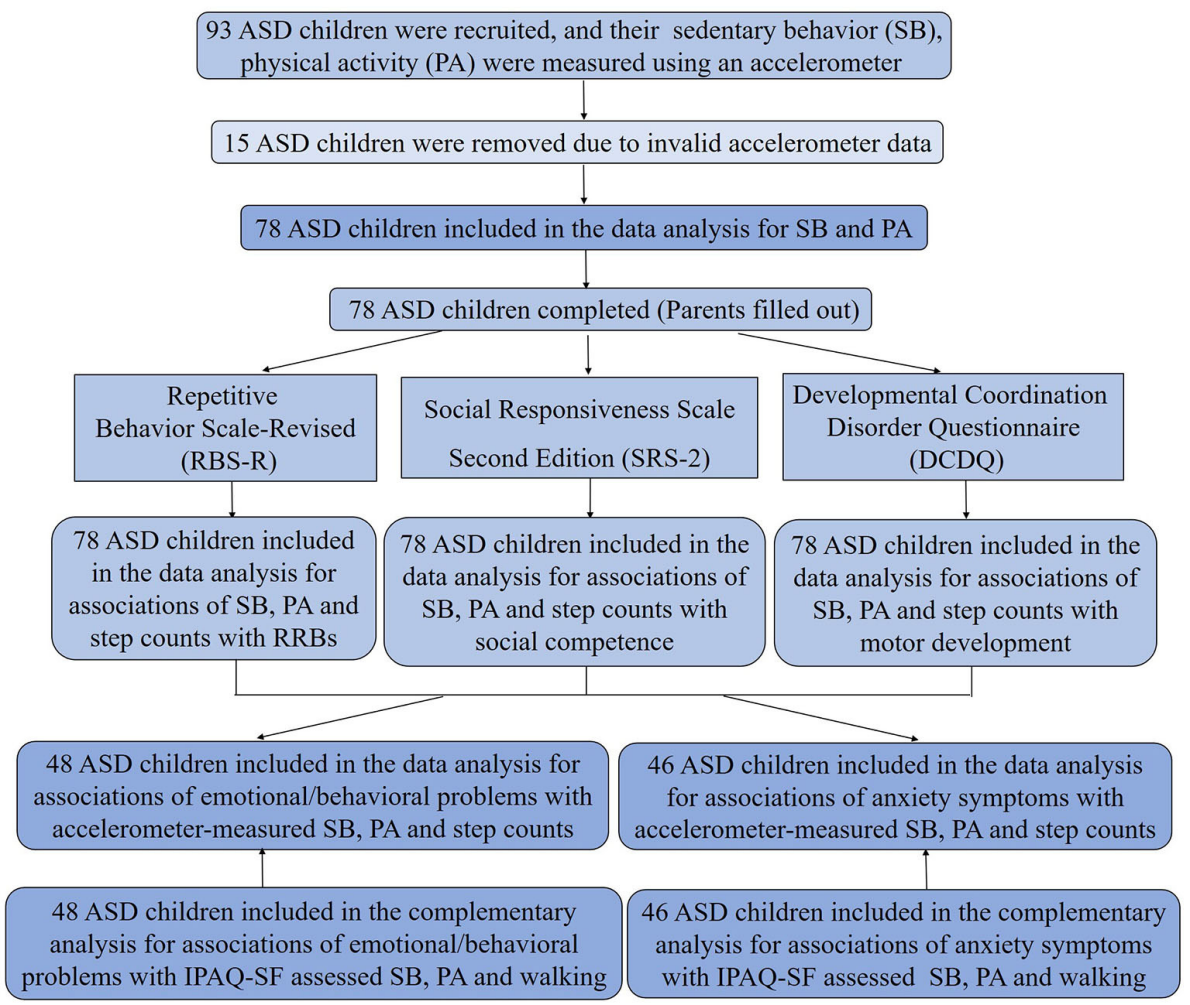
Flow diagram of study participant enrollment. IPAQ-SF, International Physical Activity Questionnaire-Short Form.

Following informed consent, parents were invited to fill a basic demographic and assessment questionnaire during their waiting for child to be evaluated. To measure RRBs, social defects and motor development which may affect PA levels, the parent-completed assessment questionnaires included Repetitive Behavior Scale-Revised (32), Social Responsiveness Scale Second Edition (33) and the Developmental Coordination Disorder Questionnaire (34, 35). Enrolled ASD children were scheduled for wearing an accelerometer on the 1st day after assessment. Meanwhile, parents were provided with the Strengths and Difficulties Questionnaire (SDQ) (36, 37) and Screen for Child Anxiety Related Emotional Disorders (SCARED) (38, 39) to record child's emotional/behavioral problems. Also, International Physical Activity Questionnaire-Short Form (IPAQ-SF) (40, 41) was handed out to parents for complementary recording of child's SB and PA. After children were monitored for seven consecutive days, the PA accelerometer devices, SDQ, SCARED and IPAQ-SF scales were returned to the research group by prepaid delivery service. Finally, in 78 valid accelerometer data, 48 effective SDQ and 46 effective SCARED data were obtained (Figure 1).

### Restricted repetitive behaviors

Repetitive Behavior Scale-Revised (RBS-R) is a parent or caregiver-report scale for assessing the type and severity of RRBs exhibited by the individuals with ASD. It consisted of 43 items organized into 6 subscales, each corresponding to one of the principal typologies of RRBs: stereotypic behavior, self-injurious behavior (SIB), compulsive behavior, ritualistic behavior, sameness behavior, and restricted interests. Each item is scored on a 0-point (never) to 3-point (always) Likert-type scale; therefore, higher scores indicate more severe RRBs. The RBS-R has been verified to good internal consistency, retest reliability, and discriminant validity (32).

### Social competence

Social Responsiveness Scale Second Edition (SRS-2) is a parent-, teacher-, or caregiver-report quantitative measurement of the presence and severity of social deficits (past 6 months) in children aged 4 to 18 years with ASD. The SRS-2 consists of 65-item, and each item is on a 4-point scale from “0” (never) to “3” (always). It generates a total composite score and subscale scores for social awareness, social cognition, social communication, social motivation, and autistic mannerisms. Higher SRS-2 scores indicate more problematic social behaviors. The scale takes a quantitative approach to measuring autistic traits or broader autism symptomology across the entire range of severity that occurs in nature. It showed 0.85 sensitivity and 0.75 specificity for identifying clinically significant autistic traits in validation studies (33).

### Motor development

Developmental coordination disorder questionnaire (DCDQ) is a parent-reported questionnaire designed to identify developmental coordination disorder (DCD) in children from 8 years to 14 years 6 months of age. The questionnaire has high internal consistence with a Cronbach's alpha coefficient of 0.88 (34). The validated Chinese version of 17 items of motor development for children aged 6 to 9 years was used in the present study (35). Parents were asked to compare their child's motor performance to that of his/her peers using a 5-point Likert-type scale. The DCDQ provides a total score (17–85 points) as well as splits scores for three results: “an indication of DCD” (≤ 48 points), “suspect DCD” (49–57 points), and “probably not DCD” (≥58 points) (34).

### Emotional and behavioral outcomes

The SDQ is designed to identify and quantify children's emotional/behavioral problems in the past 6 months. The SDQ (age range, 4–16 years) has shown satisfactory psychometric properties confirming it as a valuable tool for detecting psychosocial functioning (36). This questionnaire comprises 25 items, and each item is rated by a 3-point scale (0 = not true, 1 = somewhat true, 2 = certainly true). Every 5 out of the 25 items form a domain, adding up to a total of 5 domains: emotional symptoms, conduct problems, hyperactivity-inattention, peer problems and prosocial behavior scored from 0 to 10 each. A total difficulties score (ranging 0–40) was generated by summing all domains scores except prosocial behavior. High scores signify more unfavorable problems, except for prosocial behavior, for which a higher score indicates a more favorable outcome. The SDQ score can also be used as a categorical variable into three groups of “Normal,” “Borderline,” and “Abnormal” by standard cutoffs (37).

### Anxiety outcomes

Anxiety is a common abnormal emotion state (42), and thus the SCARED as a measure of anxiety symptoms was utilized to assess anxiety levels in youth aged 9–18 years within the past 6 months. The SCARED has demonstrated excellent discriminant validity, concurrent validity, retest reliability and internal consistence (38). We used the Chinese version revised by Chen completed by parents and can be used with children ages 6–12 years (39). The questionnaire contains 26 items assigned to four-dimensional symptoms including social phobia, separation anxiety, panic/somatic, and generalized anxiety. Every item was scored on a four-point Likert-type scale, and the items were summed to produce an overall anxiety score as well as subscale scores with higher scores indicating greater anxiety. Normal range for the norms of subscale and total score of the revised SCARED can be represented as mean ± standard deviation ($\bar{x}$ ± SD), which ≥ $\bar{x}$ + SD manifesting the presence of anxiety symptoms.

### IPAQ-SF measured SB and PA

The reliability and validity of IPAQ-SF have been verified across diverse populations (40). The Chinese version of IPAQ-SF used in the present study is originally translated by Macfarlane et al. (41), and was specially used for complementary monitoring of child's SB and PA in our study to further verify the associations of emotional symptoms with SB, PA and walking. Parents were asked to record the number of days children performed each activity (frequency) and the length of time (duration) children were involved in each daily activity for seven consecutive days of accelerometer wear, as well as the average time spent in SB. The results were used to estimate the amount of time spent in PA per day, expressed in Metabolic Equivalent of Task-minutes per day (MET-min/day). According to the official IPAQ-SF scoring protocol, PA was categorized into three different intensity levels: moderate (4 METs), vigorous (8 METs) and walking (3.3 METs) (43, 44).

### Statistical analyses

The normal distributions of continuous variables were evaluated by Kolmogorov-Smirnov (K-S) test. The continuous variables were expressed as median ± interquartile range and compared between subgroups applying the Mann-Whitney U test due to a skewed distribution. The categorical variables were expressed as number (percentage) and compared between subgroups by using the chi-square test. Multivariate generalized linear models were performed to determine unstandardized regression coefficient β with 95% confidence interval (CI) for the crude and adjusted associations of SDQ and SCARED continuous outcomes with SB, PA and step counts, and the associations of accelerometer-measured SB, PA and step counts with RRBs, poor social competence and motor development restrictions. The logistic regression models generated odds ratio (OR) with 95% CI were adopted to study the crude and adjusted associations of binary SDQ and SCARED outcomes with SB, PA and step counts, and the associations between PAG of ≥60 min/d in MVPA with RRBs, poor social competence and motor development restrictions. The five SDQ subscales and overall SDQ score were analyzed as a dichotomized variable into two groups: Borderline/Abnormal vs. Normal. The four SCARED subscale scores and the overall anxiety score were divided into asymptomatic and symptomatic groups.

To limit potential bias in the presence of covariates, information about child-, mother-, and family-level covariates was gathered through completing a sociodemographic questionnaire by participant's parents. Child-related covariates included: age (years), gender (girls or boys), severity of ASD symptoms (mild-to-moderate, severe), intellectual functioning (FSIQ < 70, FSIQ ≥ 70), intervention training (yes or no), accelerometer wear season (spring or summer) and daily accelerometer wear time (hours). Mother-level covariates incorporated: maternal age (< 35 years, ≥35 years), maternal educational level (junior college or below, university or above). A family-specific covariate was monthly per-capita income (<5,000 Renminbi “RMB,” 5,000–7,999 RMB, 8,000–11,999 RMB, or ≥ 12,000 RMB). Of the possible covariates, two covariates (intervention training and accelerometer wear season) were neither associated with dependent variables nor with independent variables (for more details see the related description of the Results section in **Table 2** and Supplementary Table 1), and therefore these two variables were excluded from a set of covariates, and the remaining covariates adjusted in all models included children's age, gender, severity of ASD symptoms, intellectual functioning, daily accelerometer wear time, maternal age, maternal educational level and monthly per-capita income. Collinearity diagnosis was performed, and there was no multicollinearity among all the covariates and independent variables included in the models.

In our study, the statistical power (1-β err prob) for multiple linear regression was calculated as 0.84 by using G^*^Power 3.1 software (45). The significance level for all analyses was set at a two-tailed *p*-values < 0.05. Data were analyzed using SPSS Statistics 26.0 (IBM Corp. Released 2019. IBM SPSS Statistics for Windows, Version 26.0. Armonk, NY, USA: IBM Corp).

## Results

### Study participants

The descriptive characteristics of 78 participants were presented in Table 1. The median age was 7.0 years, 68 (87.2%) were boys, 54 (69.2%) had mild-to-moderate ASD symptoms, 34 (43.6%) of FSIQ <70, 35 (44.9%) accepted intervention training and 20 (25.6%) wore an accelerometer in spring. Maternal age distribution was < 35 years, *n* = 25 (32.1%). Maternal educational level, junior college or below, *n* = 42 (53.8%). Monthly per-capita income was categorized into <5,000 RMB, *n* = 29 (37.2%); 5,000–7,999 RMB, *n* = 23 (29.5%); 8,000–11,999 RMB, *n* = 13 (16.7%); ≥12,000 RMB, *n* = 13 (16.7%).

**Table 1:** Descriptive characteristics of the participants, their mothers and families.

**Characteristics**	**Overall (*n* = 78)**
**Child characteristics**	
**Age, years (median ±IQR)**	7.0 ± 2.0
**Gender**, ***n*** **(%)**	
Male	68 (87.2)
Female	10 (12.8)
**Severity of ASD symptoms**^a^, ***n*** **(%)**	
Mild-to-moderate	54 (69.2)
Severe	24 (30.8)
**Intellectual functioning**^b^, ***n*** **(%)**	
FSIQ < 70	34 (43.6)
FSIQ ≥70	44 (56.4)
**Intervention training**, ***n*** **(%)**	
Yes	35 (44.9)
No	43 (55.1)
**Accelerometer wear season**, ***n*** **(%)**	
Spring	20 (25.6)
Summer	58 (74.4)
**Daily accelerometer wear time, hours (median ±IQR)**	12.7 ± 1.2
**Maternal characteristics**	
**Maternal age**, ***n*** **(%)**	
<35 years	25 (32.1)
≥35 years	53 (67.9)
**Maternal educational level**, ***n*** **(%)**	
Junior college or below	42 (53.8)
University or above	36 (46.2)
**Family characteristics**	
**Monthly per-capita income**, ***n*** **(%)**	
<5,000 RMB	29 (37.2)
5,000–7,999 RMB	23 (29.5)
8,000–11,999 RMB	13 (16.7)
≥12,000 RMB	13 (16.7)

ASD, autism spectrum disorder; FSIQ, full-scale intelligence quotient; IQR, interquartile range; RMB, Renminbi.

a Assessment by Childhood Autism Rating Scale (CARS).

b Assessment by Wechsler Intelligence Scale for Children 4th Edition (WISC-IV).

### SB, PA, and step counts in children with ASD

The median daily duration of SB, PA and step counts were presented in Table 2. Of the 78 participants, daily SB, LPA, MPA, VPA, MVPA, and total PA (LPA, MPA and VPA) averaged 6.99 h, 290.18 min, 36.15, 15.62, 51.95 and 346.71 min, respectively. The percentage of participants meeting the PAG of 60 min of MVPA per day was 37.2%. As for step counts, participants averaged of 9,560 steps per day and 12.00 steps per minute.

**Table 2:** Sedentary behavior, physical activity, and step counts in children with ASD (*n* = 78).

**Variables**	**Overall** **(*n* = 78)**	**Mild-to-moderate ASD** **(*n* = 54)**	**Severe ASD** **(*n* = 24)**	**FSIQ < 70** **(*n* = 34)**	**FSIQ ≥70** **(*n* = 44)**	**Intervention training** **(*n* = 35)**	**No intervention training** ** (*n* = 43)**	**Measuring in spring** **(*n* = 20)**	**Measuring in summer** **(*n* = 58)**
**SB variables**									
SB, hours/d	6.99 ± 1.50	7.16 ± 1.84	6.58 ± 1.41	6.58 ± 1.51	7.21 ± 1.84^*^	7.16 ± 1.91	6.89 ± 1.47	6.93 ± 2.27	7.04 ± 1.51
**PA variables**									
LPA, min/d	290.18 ± 64.71	288.84 ± 61.13	293.35 ± 63.56	299.21 ± 68.08	283.36 ± 58.36	284.39 ± 53.86	296.00 ± 80.18	299.41 ± 66.05	288.39 ± 68.57
MPA, min/d	36.15 ± 22.05	35.73 ± 20.17	37.06 ± 21.80	38.93 ± 20.58	34.11 ± 18.42	35.14 ± 19.68	37.20 ± 23.29	33.95 ± 22.56	36.81 ± 20.41
VPA, min/d	15.62 ± 15.56	12.05 ± 16.37	21.26 ± 17.95^*^	21.18 ± 15.66	11.03 ± 16.27^*^	11.68 ± 17.03	18.14 ± 15.03	16.13 ± 17.77	15.62 ± 15.37
MVPA, min/d	51.95 ± 37.78	49.11 ± 35.05	60.53 ± 35.86	60.53 ± 32.56	47.21 ± 28.30^*^	48.46 ± 34.75	54.25 ± 37.06	54.23 ± 43.72	51.54 ± 35.59
MVPA ≥ 60 min/d	29 (37.2)	17 (31.5)	12 (50.0)	17 (50.0)	12 (27.3)^*^	11 (31.4)	18 (41.9)	7 (35.0)	22 (37.9)
Total PA, min/d	346.71 ± 80.27	336.41 ± 73.24	370.63 ± 81.45	374.30 ± 85.91	330.26 ± 70.63	339.63 ± 76.95	353.79 ± 83.46	350.64 ± 75.90	346.29 ± 82.11
Thousand steps/d	9.56 ± 2.91	9.34 ± 2.97	10.55 ± 4.05	10.22 ± 3.87	9.38 ± 2.63	9.45 ± 4.18	9.61 ± 2.52	9.79 ± 2.90	9.56 ± 3.31
Steps/min	12.20 ± 4.03	12.05 ± 3.95	13.45 ± 5.67	13.15 ± 4.93	11.90 ± 3.82^*^	13.10 ± 5.20	12.20 ± 3.80	12.25 ± 4.28	12.20 ± 4.03

ASD, autism spectrum disorder; SB, sedentary behavior; PA, physical activity; LPA, low PA; MPA, moderate PA; VPA, vigorous PA; MVPA, moderate-to-vigorous PA; FSIQ, full-scale intelligence quotient; IQR, interquartile range.

Data were reported as median ± IQR or numbers with percentages. Mann-Whitney U and chi-square tests were used to compare variables between subgroups. Total PA included LPA, MPA, and VPA. The intervention trainings included the behavioral interventions, educational interventions, social interventions, and the integrated intervention model.

* Statistically significant differences between subgroups of mild-to-moderate and severe ASD; subgroups of FSIQ <70 and FSIQ ≥70; subgroups of intervention training; subgroups of accelerometer wear season (p < 0.05).

Average daily duration of SB, PA variables and step counts across different subgroups were also displayed in Table 2. When analyzed according to the severity symptoms, the subgroup of mild-to-moderate ASD spent less daily VPA time than severe ASD (*p* = 0.038). There were substantial differences in average daily time for SB, VPA, MVPA and step counts between FSIQ ≥ 70 and FSIQ < 70 (*p* < 0.05). Specifically, the SB hours in FSIQ ≥ 70 were longer than in FSIQ < 70 (*p* = 0.027). Inversely, the average minutes of VPA and MVPA were shorter, as well as steps per minute were less in FSIQ ≥ 70 than FSIQ < 70 (*p* = 0.009; *p* = 0.029; *p* = 0.048). The relatively lower proportion of conforming to the PAG was observed in subgroup of FSIQ ≥ 70 (*p* = 0.020). There were no significant differences for SB, PA variables and step counts between the intervention training or not, accelerometer wearing in spring or summer (Table 2). Based on these findings, the SDQ and SCARED scores were compared between the subgroups of intervention training, the subgroups of accelerometer wear season, and no significant differences were observed (see Supplementary Table 1).

### Associations between accelerometer-measured SB, PA, and step counts and ASD-related deficiencies in children with ASD

The findings of present study demonstrated that accelerometer-measured SB, PA and step counts had no significant associations with ASD-related deficiencies including RRBs, poor social competence and motor development restrictions (*p* > 0.05) (Supplementary Tables 2–4). With these results, the associations of emotional/behavioral problems with SB, PA and step counts are worthy of being explored.

### Associations of emotional/behavioral problems (SDQ) with accelerometer-measured SB, PA, and step counts in children with ASD

The associations between continuous SDQ scores and SB, PA, and step counts were presented in Table 3. Overall, SDQ emotional symptoms were inversely associated with the average daily VPA duration in crude (β = −0.075, p = 0.002, 95% CI [−0.121, −0.028]) and adjusted (β = −0.060, *p* = 0.028, 95% CI [−0.114, −0.006]) models. Additionally, the inverse association between emotional symptoms and daily MVPA duration (β = −0.033, *p* = 0.016, 95% CI [−0.059, −0.006]) was significant for ASD in the crude model.

**Table 3:** Associations of the levels of emotional/behavioral problems (SDQ) with accelerometer-measured SB, PA and step counts in children with ASD (*n* = 48).

**Variables**	β **coefficient (95% confidence interval)**					
	**Emotional symptoms**	**Conduct problems**	**Hyperactivity-inattention**	**Peer problems**	**Prosocial behavior**	**Total difficulties**
**SB (hours/d)**						
Crude model	0.461 (−0.038, 0.960)	0.446 (0.104, 0.788)^*^	−0.227 (−0.855, 0.401)	−0.290 (−0.775, 0.196)	0.186 (−0.412, 0.785)	0.390 (−0.879, 1.660)
Adjusted model	0.538 (−0.138, 1.215)	0.274 (−0.158, 0.706)	−0.502 (−1.331, 0.327)	−0.082 (−0.695, 0.532)	−0.296 (−0.964, 0.373)	0.229 (−1.569, 2.026)
**LPA (min/d)**						
Crude model	0.000 (−0.013, 0.012)	−0.008 (−0.016, 0.001)	0.012 (−0.003, 0.027)	0.008 (−0.004, 0.019)	0.000 (−0.015, 0.014)	0.011 (−0.019, 0.042)
Adjusted model	−0.006 (−0.019, 0.006)	−0.005 (−0.013, 0.003)	0.007 (−0.008, 0.023)	0.003 (−0.008, 0.014)	0.005 (−0.007, 0.018)	−0.001 (−0.034, 0.033)
**MPA (min/d)**						
Crude model	−0.030 (−0.078, 0.018)	−0.005 (−0.040, 0.029)	0.029 (−0.030, 0.088)	0.000 (−0.046, 0.047)	−0.011 (−0.067, 0.046)	−0.006 (−0.126, 0.114)
Adjusted model	−0.022 (−0.070, 0.026)	−0.011 (−0.041, 0.020)	0.013 (−0.045, 0.072)	−0.005 (−0.048, 0.037)	0.002 (−0.045, 0.049)	−0.025 (−0.151, 0.100)
**VPA (min/d)**						
Crude model	−0.075 (−0.121, −0.028)^**^	−0.016 (−0.052, 0.020)	0.048 (−0.013, 0.109)	0.024 (−0.024, 0.073)	−0.052 (−0.109, 0.006)	−0.018 (−0.145, 0.108)
Adjusted model	−0.060 (−0.114, −0.006)^*^	−0.006 (−0.041, 0.030)	0.044 (−0.023, 0.111)	−0.019 (−0.068, 0.031)	0.016 (−0.039, 0.070)	−0.041 (−0.186, 0.105)
**MVPA (min/d)**						
Crude model	−0.033 (−0.059, −0.006)^*^	−0.007 (−0.026, 0.013)	0.024 (−0.010, 0.058)	0.008 (−0.019, 0.034)	−0.019 (−0.052, 0.013)	−0.008 (−0.077, 0.062)
Adjusted model	−0.024 (−0.053, 0.004)	−0.005 (−0.024, 0.013)	0.017 (−0.018, 0.052)	−0.007 (−0.033, 0.019)	0.005 (−0.024, 0.033)	−0.020 (−0.096, 0.055)
**MVPA ≥60 min/d** ^a^						
Crude model	−1.139 (−2.300, 0.023)	−0.408 (−1.252, 0.437)	1.134 (−0.304, 2.573)	0.101 (−1.049, 1.251)	−0.819 (−2.203,0.564)	−0.311 (−3.286, 2.664)
Adjusted model	−0.675 (−1.954, 0.603)	−0.347 (−1.159, 0.465)	0.759 (−0.794, 2.311)	−0.549 (−1.684, 0.585)	0.245 (−1.009, 1.500)	−0.813 (−4.159, 2.532)
**Total PA (min/d)**						
Crude model	−0.005 (−0.017, 0.006)	−0.459 (−0.920, 0.003)	0.014 (0.001, 0.027)^*^	0.008 (−0.003, 0.018)	−0.003 (−0.017, 0.010)	0.008 (−0.020, 0.036)
Adjusted model	−0.009 (−0.020, 0.002)	−0.005 (−0.012, 0.003)	0.008 (−0.005, 0.022)	0.001 (−0.009, 0.012)	0.005 (−0.006, 0.016)	−0.004 (−0.034, 0.026)
**Thousand steps/d**						
Crude model	−0.081 (−0.300, 0.138)	−0.011 (−0.167, 0.144)	0.141 (−0.125, 0.408)	0.100 (−0.108, 0.308)	0.000 (−0.257, 0.256)	0.149 (−0.393, 0.692)
Adjusted model	−0.120 (−0.357, 0.118)	−0.010 (−0.162, 0.141)	0.079 (−0.211, 0.369)	0.012 (−0.201, 0.224)	0.098 (−0.133, 0.330)	−0.040 (−0.662, 0.583)
**Steps/min**						
Crude model	−0.084 (−0.241, 0.073)	−0.030 (−0.142, 0.082)	0.075 (−0.119, 0.268)	0.071 (−0.080, 0.221)	−0.007 (−0.192, 0.178)	0.031 (−0.361, 0.423)
Adjusted model	−0.096 (−0.275, 0.082)	−0.016 (−0.130, 0.098)	0.059 (−0.159, 0.277)	0.003 (−0.157, 0.163)	0.076 (−0.098, 0.250)	−0.050 (−0.518, 0.418)

SDQ, strengths and difficulties questionnaire; ASD, autism spectrum disorder; SB, sedentary behavior; PA, physical activity; LPA, low PA; MPA, moderate PA; VPA, vigorous PA; MVPA, moderate-to-vigorous PA.

Crude model: no adjust. Adjusted model: adjusted for age, gender, severity of ASD symptoms, intellectual functioning, daily accelerometer wear time, maternal age, maternal educational level and monthly per-capita income.

a Compared to MVPA < 60 min/d.

* Statistically significant associations (p < 0.05);

** Statistically significant associations (p < 0.01).

When the dependent variable SDQ score was modeled as a dichotomous variable (Table 4), the lower odds of emotional symptoms were significantly associated with daily duration of VPA (OR = 0.829, *p* = 0.005, 95% CI [0.727, 0.946]) in crude model. The adjusted logistic regression exhibited the same associations between emotional symptoms and VPA (OR = 0.820, *p* = 0.008, 95% CI [0.708, 0.950]). A significantly lowed odds of emotional symptoms with a duration of MVPA was also observed in both the crude (OR = 0.943, *p* = 0.012, 95% CI [0.901, 0.987]) and adjusted (OR = 0.937, *p* = 0.018, 95% CI [0.888, 0.989]) models. Participants who met PAG showed lower likelihood of emotional symptoms relative to those who did not in both crude (OR = 0.110, *p* = 0.044, 95% CI [0.013, 0.939]) and adjusted (OR = 0.057, *p* = 0.034, 95% CI [0.004, 0.810]) regression models.

**Table 4:** Associations of emotional/behavioral problems (SDQ) with accelerometer-measured SB, PA, and step counts in children with ASD (*n* = 48).

**Variables**	**Odds ratio (95% confidence interval)**					
	**Emotional symptoms**	**Conduct problems**	**Hyperactivity-inattention**	**Peer problems**	**Prosocial behavior**	**Total difficulties**
**SB (hours/d)**						
Crude model	1.470 (0.802, 2.696)	1.735 (0.939, 3.208)	1.005 (0.552, 1.830)	1.191 (0.455, 3.116)	1.043 (0.573, 1.899)	1.187 (0.615, 2.289)
Adjusted model	2.142 (0.809, 5.673)	0.953 (0.259, 3.499)	0.880 (0.302, 2.568)	0.746 (0.064, 8.665)	0.885 (0.288, 2.723)	1.100 (0.385, 3.146)
**LPA (min/d)**						
Crude model	1.002 (0.988, 1.016)	0.991 (0.977, 1.006)	1.007 (0.992, 1.023)	1.014 (0.986, 1.044)	1.003 (0.988, 1.018)	0.997 (0.982, 1.013)
Adjusted model	0.993 (0.976, 1.011)	1.001 (0.978, 1.024)	0.999 (0.980, 1.019)	1.008 (0.957, 1.062)	1.000 (0.980, 1.021)	0.994 (0.975, 1.014)
**MPA (min/d)**						
Crude model	0.954 (0.899, 1.013)	1.009 (0.958, 1.063)	1.013 (0.956, 1.073)	1.050 (0.944, 1.167)	0.996 (0.941, 1.053)	1.026 (0.962, 1.096)
Adjusted model	0.944 (0.876, 1.017)	1.008 (0.931, 1.091)	1.017 (0.934, 1.085)	0.975 (0.819, 1.160)	1.016 (0.942, 1.096)	1.021 (0.940, 1.110)
**VPA (min/d)**						
Crude model	0.829 (0.727, 0.946)^**^	0.994 (0.940, 1.051)	1.041 (0.971, 1.116)	1.062 (0.930, 1.212)	1.024 (0.960, 1.093)	1.049 (0.968, 1.138)
Adjusted model	0.820 (0.708, 0.950)^**^	0.995 (0.916, 1.081)	1.076 (0.959, 1.208)	1.089 (0.764, 1.551)	1.020 (0.934, 1.114)	1.083 (0.961, 1.221)
**MVPA (min/d)**						
Crude model	0.943 (0.901, 0.987)^*^	1.001 (0.971, 1.032)	1.016 (0.981, 1.052)	1.035 (0.968, 1.108)	1.005 (0.972, 1.039)	1.023 (0.982, 1.066)
Adjusted model	0.937 (0.888, 0.989)^*^	1.001 (0.957, 1.047)	1.021 (0.969, 1.075)	0.999 (0.886, 1.125)	1.012 (0.966, 1.060)	1.030 (0.973, 1.091)
**MVPA ≥60 min/d** ^a^						
Crude model	0.110 (0.013, 0.939)^*^	1.019 (0.278, 3.737)	1.528 (0.350, 6.674)	1.258 (0.120, 13.245)	1.528 (0.350, 6.674)	4.680 (0.533, 41.069)
Adjusted model	0.057 (0.004, 0.810)^*^	1.094 (0.139, 8.630)	1.271 (0.184, 8.790)	0.213 (0.003, 14.213)	1.091 (0.143, 8.323)	4.025 (0.317, 51.169)
**Total PA (min/d)**						
Crude model	0.995 (0.982, 1.008)	0.993 (0.981, 1.006)	1.008 (0.994, 1.023)	1.018 (0.990, 1.047)	1.003 (0.990, 1.017)	1.001 (0.987, 1.015)
Adjusted model	0.987 (0.971, 1.004)	1.001 (0.979, 1.023)	1.002 (0.984, 1.020)	1.005 (0.965, 1.047)	1.002 (0.983, 1.021)	0.998 (0.981, 1.016)
**Thousand steps/d**						
Crude model	0.875 (0.676, 1.133)	1.067 (0.841, 1.353)	1.054 (0.813, 1.366)	1.112 (0.722, 1.711)	0.840 (0.646, 1.090)	1.015 (0.766, 1.344)
Adjusted model	0.836 (0.594, 1.176)	1.384 (0.831, 2.305)	1.002 (0.685, 1.466)	0.675 (0.195, 2.334)	0.950 (0.651, 1.387)	1.028 (0.685, 1.543)
**Steps/min**						
Crude model	0.887 (0.730, 1.078)	1.014 (0.854, 1.203)	1.010 (0.839, 1.216)	1.009 (0.748, 1.361)	0.874 (0.727, 1.052)	0.987 (0.808, 1.206)
Adjusted model	0.867 (0.670, 1.123)	1.248 (0.856, 1.819)	1.000 (0.752, 1.331)	0.678 (0.228, 2.019)	0.961 (0.723, 1.277)	1.018 (0.751, 1.379)

SDQ, Strengths and difficulties questionnaire; ASD, autism spectrum disorder; SB, sedentary behavior; PA, physical activity; LPA, low PA; MPA, moderate PA; VPA, vigorous PA; MVPA, moderate-to-vigorous PA.

Crude model: no adjust. Adjusted model: adjusted for age, gender, severity of ASD symptoms, intellectual functioning, daily accelerometer wear time, maternal age, maternal educational level and monthly per-capita income.

a Compared to MVPA < 60 min/d.

* Statistically significant associations (p < 0.05);

** Statistically significant associations (p < 0.01).

### Associations of emotional/behavioral problems (SDQ) with IPAQ-SF assessed SB, PA, and walking in children with ASD

When the data were obtained from IPAQ-SF, we identified the consistent inverse associations of emotional symptoms with VPA (β = −0.018, p = 0.010, 95% CI [−0.031, −0.004]) and MVPA (β = −0.009, *p* = 0.009, 95% CI [−0.017, −0.002]), respectively, in crude models. We also discovered the inverse associations of emotional symptoms with VPA (β = −0.020, *p* = 0.004, 95% CI [−0.033, −0.006]) and MVPA (β = −0.011, *p* = 0.003, 95% CI [−0.018, −0.004]), respectively, after covariate-adjustment (Supplementary Table 6).

When the dependent variable was dichotomous, lower odds of emotional symptoms were associated with more VPA (OR = 0.966, *p* =0.039, 95% CI [0.935, 0.998]) and MVPA (OR = 0.979, *p* = 0.041, 95% CI [0.958, 0.999]), respectively, in adjusted models (Supplementary Table 7).

### Associations of anxiety symptoms (SCARED) with accelerometer-measured SB, PA, and step counts in children with ASD

The associations between continuous SCARED scores and SB, PA and step counts were exhibited in Table 5. After adjustment for the covariates, SCARED somatic/panic was inversely associated with the average daily VPA minutes (β = −0.007, *p* = 0.040, 95% CI [−0.013, 0.000]) and generalized anxiety levels were also inversely associated with the average daily VPA minutes (β = −0.025, *p* = 0.014, 95% CI [−0.045, −0.005]). Additionally, SCARED total anxiety levels were conversely associated with the daily MVPA minutes (β = −0.006, *p* = 0.029, 95% CI [−0.011, −0.001]). A lower level of social phobia was associated with ≥60 min/d of MVPA in comparison to < 60 min/d (β = −0.391, *p* = 0.047, 95% CI [−0.775, −0.006]).

**Table 5:** Associations of anxiety levels (SCARED) with accelerometer-measured SB, PA, and step counts in children with ASD (*n* = 46).

**Variables**	β **coefficient (95% confidence interval)**				
	**Social phobia**	**Separation anxiety**	**Somatic/panic**	**Generalized anxiety**	**Total scores**
**SB (hours/d)**					
Crude model	0.182 (0.043, 0.321)^*^	0.052 (−0.025, 0.129)	0.054 (0.002, 0.106)^*^	0.118 (−0.043, 0.278)	0.113 (0.032, 0.194)^*^
Adjusted model	0.142 (−0.037, 0.322)	0.009 (−0.092, 0.111)	0.004 (−0.061, 0.068)	0.071 (−0.134, 0.275)	0.070 (−0.039, 0.178)
**LPA (min/d)**					
Crude model	−0.002 (−0.006, 0.002)	−0.001 (−0.003, 0.001)	0.000 (−0.001, 0.001)	−0.001 (−0.005, 0.003)	−0.001 (−0.003, 0.001)
Adjusted model	−0.002 (−0.005, 0.002)	0.000 (−0.002, 0.002)	0.000 (−0.001, 0.001)	0.000 (−0.004, 0.004)	−0.001 (−0.003, 0.001)
**MPA (min/d)**					
Crude model	−0.012 (−0.026, 0.002)	−0.005 (−0.012, 0.003)	−0.004 (−0.009, 0.001)	−0.006 (−0.022, 0.009)	−0.008 (−0.016, 0.001)
Adjusted model	−0.015 (−0.030, −0.001)^*^	−0.005 (−0.013, 0.003)	−0.002 (−0.007, 0.004)	−0.010 (−0.027, 0.007)	−0.009 (−0.018, 0.000)^*^
**VPA (min/d)**					
Crude model	−0.004 (−0.022, 0.014)	0.001 (−0.009, 0.010)	−0.005 (−0.011, 0.002)	−0.027 (−0.046, −0.008)^*^	−0.006 (−0.017, 0.004)
Adjusted model	−0.012 (−0.031, 0.007)	−0.004 (−0.015, 0.006)	−0.007 (−0.013, 0.000)^*^	−0.025 (−0.045, −0.005)^*^	−0.011 (−0.022, 0.000)
**MVPA (min/d)**					
Crude model	−0.006 (−0.014, 0.003)	−0.002 (−0.006, 0.003)	−0.003 (−0.006, 0.001)	−0.009 (−0.019, 0.001)	−0.004 (−0.010, 0.001)
Adjusted model	−0.008 (−0.017, 0.000)	−0.003 (−0.008, 0.002)	−0.002 (−0.005, 0.001)	−0.009 (−0.019, 0.001)	−0.006 (−0.011, −0.001)^*^
**MVPA ≥60 min/d** ^a^					
Crude model	−0.239 (−0.641, 0.163)	−0.008 (−0.224, 0.207)	−0.067 (−0.216, 0.081)	−0.329 (−0.769, 0.111)	−0.155 (−0.391, 0.080)
Adjusted model	−0.391 (−0.775, −0.006)^*^	−0.039 (−0.261, 0.182)	−0.065 (−0.205, 0.075)	−0.337 (−0.775, 0.100)	−0.221 (−0.452, 0.010)
**Total PA (min/d)**					
Crude model	−0.002 (−0.005, 0.001)	−0.038 (−0.139, 0.063)	0.000 (−0.001, 0.001)	−0.002 (−0.005, 0.002)	−0.001 (−0.003, 0.000)
Adjusted model	−0.002 (−0.005, 0.001)	0.000 (−0.002, 0.002)	0.000 (−0.001, 0.001)	−0.001 (−0.005, 0.002)	−0.001 (−0.003, 0.001)
**Thousand steps/d**					
Crude model	−0.065 (−0.142, 0.013)	−0.020 (−0.062, 0.022)	−0.015 (−0.044, 0.014)	−0.047 (−0.134, 0.041)	−0.040 (−0.086, 0.006)
Adjusted model	−0.044 (−0.126, 0.039)	−0.012 (−0.058, 0.034)	−0.002 (−0.031, 0.027)	−0.021 (−0.114, 0.072)	−0.024 (−0.074, 0.025)
**Steps/min**					
Crude model	−0.057 (−0.112, −0.003)^*^	−0.015 (−0.044, 0.015)	−0.015 (−0.035, 0.006)	−0.036 (−0.097, 0.026)	−0.034 (−0.066, −0.002)^*^
Adjusted model	−0.035 (−0.096, 0.027)	−0.006 (−0.040, 0.029)	0.000 (−0.022, 0.022)	−0.011 (−0.080, 0.059)	−0.017 (−0.054, 0.020)

SCARED, screen for child anxiety related emotional disorders; ASD, autism spectrum disorder; SB, sedentary behavior; PA, physical activity; LPA, low PA; MPA, moderate PA; VPA, vigorous PA; MVPA, moderate-to-vigorous PA.

Crude model: no adjust. Adjusted model: adjusted for age, gender, severity of ASD symptoms, intellectual functioning, daily accelerometer wear time, maternal age, maternal educational level and monthly per-capita income.

a Compared to MVPA < 60 min/d.

* Statistically significant associations (p < 0.05).

When SCARED score modeled as a dichotomous variable, decreased odds of generalized anxiety was significantly associated with more accelerometer assessed VPA (OR = 0.928, *p* = 0.033, 95% CI [0.866, 0.994]) in the crude model, see Supplementary Table 5.

Moreover, SCARED social phobia (β = −0.015, *p* = 0.040, 95% CI [−0.030, −0.001]) and total anxiety levels (β = −0.009, *p* = 0.043, 95% CI [−0.018, 0.000]) were inversely associated with the average daily MPA minutes as in Table 5.

### Associations of anxiety symptoms (SCARED) with IPAQ-SF assessed SB, PA, and walking in children with ASD

When the data were obtained from IPAQ-SF, adjusted negative associations of social phobia (β = −0.006, *p* = 0.010, 95% CI [−0.011, −0.001]) with VPA, total anxiety levels (β = −0.003, *p* = 0.014, 95% CI [−0.006, −0.001]) with VPA were identified, respectively, see Supplementary Table 8. When SCARED score modeled as a dichotomous variable, no significant associations of anxiety symptoms with IPAQ-SF assessed SB, PA and walking were found, see Supplementary Table 9.

### Associations of emotional symptoms with accelerometer-measured PA in mild-to-moderate and severe ASD children

The associations of emotional symptoms with VPA and MVPA were analyzed in mild-to-moderate and severe ASD children, respectively. After adjusting for covariates, there existed significantly negative associations of emotional symptoms with VPA (β = −0.087, *p* = 0.029, 95% CI [−0.166, −0.009]) in mild-to-moderate ASD children. Also, there existed significantly negative associations of emotional symptoms with VPA (β = −0.129, *p* < 0.001, 95% CI [−0.165, −0.094]) and MVPA (β = −0.074, *p* < 0.001, 95% CI [−0.084, −0.064]) in severe ASD children, see Supplementary Table 10 for further details.

## Discussion

Our findings revealed that no significant associations between accelerometer-measured SB, PA and step counts and ASD-associated deficiencies were identified. Furthermore, the consistent inverse associations of emotional symptoms with VPA and MVPA in crude and adjusted models were discovered, when we combined the accelerometer data with the IPAQ-SF data for verification. Although the subgroup of mild-to-moderate ASD spent less daily VPA time than severe ASD in our study, the adjusted negative associations between emotional symptoms and VPA remained in mild-to-moderate and severe ASD, respectively. This means that the negative association between emotional symptoms and VPA was not altered by the difference in daily VPA time between subgroups of mild-to-moderate and severe ASD. Additionally, after adjusting for covariates, the negative associations between emotional symptoms and MVPA persisted in severe ASD children, but was absent in mild-to-moderate ASD. Future studies with larger sample size are needed to better examine the associations of emotional symptoms with MVPA in mild-to-moderate ASD group. Although statistically significant negative associations with VPA and MVPA were found in one out of the SDQ five domains, the associations of the four remaining domains with VPA or MVPA were not significant: conduct problems, hyperactivity-inattention, peer problems, and prosocial behavior. The accelerometer was worn on the right hip for only 7 days. It is possible that if the accelerometer wear time had lasted for a longer period of time, these domains may show significant associations with VPA and MVPA.

### SB, PA, and step counts in children with ASD

A review published in 2020 displayed that a mean MVPA is 56.95 min/weekdays and 55.72 min/weekends in ASD individuals, respectively (46). Another review published in 2017 indicated that estimated MVPA was 34–166 min/day (47). A median daily 51.95 min of MVPA in our study was slight lower than the 56.95 and 55.72 min, but fell within the range of 34–166 min/day aforementioned. Additionally, we found that 37.2% of the participants got 60 min of accelerometer-measured MVPA daily, which was consistent with the conclusion of previous studies that < 50% of participants met the PAG of 60-min' daily MVPA (46). In contrast, other studies observed that the majority of elementary school-age children exceed the MVPA guidelines (48). Different proportions of following MVPA guidelines between studies may be attributed to the differences of children's functional ability and their school and/or home environments, leading to different time in MVPA during physical education, at recess or after school (49). For instance, Obrusnikova and Dillon (50) indicated that children with ASD were inactive in physical education (PE), and PE teachers are challenged to engage them in PA during classes. Inversely, Pan reported that ASD children are active and spent more time in MVPA during PE as compared to at recess and after school, which ascribes to the short of verbal or physical prompts from school at recess and the short of positive influence from family after school (51, 52). This suggests that teachers' and parents' involvements were major components of PA participation, and improving their support for ASD children is a viable means of enhancing MVPA.

Our data showed an average of 346.71 min total PA (LPA, MPA and VPA) per day among ASD children. It means that our study participants complied with the recommendation on children's total PA that elementary school-aged children should be physically active of at least 30 min each day to accumulate at least 210 min of overall PA each week raised by Taiwan's Ministry of Education (48). In our study, the average daily number of steps for ASD individuals was 9,560, which was smaller than BMI-referenced recommendations which suggest that 6–12 girls and boys should accumulate 12,000 and 15,000 daily steps, respectively (53). As another study reported, few elementary school-aged children could fulfill the BMI-referenced recommendations of the daily step counts (52), and this phenomenon reminds us that the related recommendation taking into account the type and intensity of PA should be reinstituted. Finally, our 78 participants spent an average of 6.99 h per day with SB. One study has reported that typically developing children (aged 3–11 years, *n* = 53) spent almost 5 h in SB per day of the week (54). This showed that ASD children spent more time in SB than typically developing children, which are supported by other studies conducted in this population (47). As the majority of parents reported that their ASD children went directly home or private nursing/talent class in the community after school and engaged in sedentary and technology-based activities that likely contributed to inactivity. Inactive lifestyles and sedentary habits are linked to high risk for developing cardiovascular diseases, diabetes and obesity (20). We therefore suggest that children with ASD should be provided with PA opportunities from school and families and social support from teachers, peers, and parents to increase PA levels.

### Associations of emotional/behavioral problems with SB, PA, and step counts in children with ASD

Given the complexity of ASD, investigating associations of SB, PA and step counts with ASD-related factors would be beneficial for assessing associations of emotional symptoms with SB, PA, and step counts. In our study, accelerometer-measured SB, PA, and step counts were not associated with ASD-related deficiencies (RRBs, poor social competence and motor development restrictions). These results suggest that ASD-related deficiencies might not be the primary effectors of SB, PA, and step counts in children with ASD. Importantly, although the associations of cognitive flexibility, peer problems, and behavioral functioning with VPA and MVPA in ASD have been reported previously (55, 56). To date, however, only few studies have used both standardized objective and subjective tests of SB, PA, and step counts and analyzed the associations of emotional symptoms with SB, PA, and step counts. Taken together, our study found that SDQ emotional symptoms were significantly and negatively associated with the average daily time spent in VPA and MVPA. In addition, we also found that SCARED somatic/panic and generalized anxiety were inversely associated with the average daily VPA minutes. The SCARED total anxiety was inversely associated with the daily MVPA duration in children with ASD. Overall, our findings imply that individuals with ASD need VPA or MVPA to exceed a particular intensity threshold value for the occurrence of positive effects. The possible reason for the threshold effect is that higher-intensity PA is more able to draw the attention away from unpleasant or painful emotional experiences (57). Thus, distraction may account for some of the antidepressant effects of acute exercise (58). Besides, studies have pointed that the presence of an optimal level of arousal modulates stimulation in an organism for maintaining sensory homeostasis, and physical exercise can facilitate arousal and stimulus modulation (59). Accordingly, we postulate that a high-intensity PA like VPA or MVPA, compared to LPA and steps, exceeding a certain stimulation threshold can better play the role of arousal to provide more appropriate sensory feedback. Another fundamental mechanism might be that VPA and MVPA may increase the rate and amount of synthesis and metabolism of neurotrophic factors, endorphins and monoamine neurotransmitters in the brain, thus mimicking the stronger effects of antidepressants and making PA an ideal strategy to manage emotional/behavioral problems with ASD (60, 61). As in one study, conducted with the same exercise frequency and duration and different exercise intensities in two groups of intervention, revealed that higher-intensity exercise group had a larger percentage decrease in serotonin than the control group (59). Decreases in serotonin was found to partially mediate the relationship between exercise and depression (62). Besides, there is the potential explanation for the observed inverse associations of emotional symptoms with VPA and MVPA that high-intensity PA can help to increase self-efficacy or sense of independence, control and success (57). Others have supported these findings, noting that increased self-efficacy was strongly related to adoption of vigorous activity (63). Previous studies suffer from several limitations, such as reliance solely on subjective measures of PA, use only one scale to assess dependent variables and failure to control for covariates in the models (64). Our study mainly used accelerometers, supplemented with IPAQ-SF to measure PA; mainly used the SDQ supplemented with SCARED to assess emotional symptoms; controlled for the covariates, and especially analyzed the associations of PA with ASD-related deficiencies. Our results indicated that emotional symptoms showed negative associations with VPA and MVPA using data from accelerometer and IPAQ-SF. Our findings, although preliminary, are encouraging since the emotional symptoms are common characteristics associated with those who have ASD. Future researches longitudinally investigating on the directionality of these associations are required. Furthermore, our findings make a promising contribution to the literature investigating the impact of different intensities of PA interventions on the emotional symptoms of children with ASD.

After stratified analysis, the negative associations between emotional symptoms and VPA remained in mild-to-moderate and severe ASD, respectively, while the negative associations between emotional symptoms and MVPA only existed in severe ASD children. Although the association of emotional symptoms with MVPA was not significant in mild-to-moderate ASD, there existed a tendency of inverse association between them. This is most likely because better emotion regulation skills possessed by mild-to-moderate ASD children (65), play a more important role than MVPA in regulating emotions. Future work should aim to clarify these complex associations.

Although numerous studies have shown the relationship between more SB and unfavorable mental health outcomes (66, 67). Our findings manifested no associations between emotional symptoms and accelerometer-measured SB. Overall, the associations between mental health outcomes and SB are rather indeterminate (67). For instance, no clear conclusion on SB and depressive symptoms, eating disorder symptoms and anxiety symptom could be drawn from several previous studies (67). The most common reasons for the inconclusive results on associations between mental health issues and SB were the existence and measurement of different types of SB (47). Many studies have used subjectively measured sedentary screen time instead of accelerometer-measured overall SB (54, 68, 69). In fact, sedentary screen time was not a suitable marker for overall SB due to the mechanisms to explain mental health issues include not only sitting time but also the negative media influences brought by screen-based SB. Hence, our results could provide higher reference values for the associations between mental health indicators and accelerometer-assessed total sitting time. Lastly, our findings showed that the SCARED social phobia and total anxiety scores exhibited opposite associations with accelerometer-measured daily MPA. However, SDQ outcome showed no significant associations with MPA. MPA involves complex, dynamic upper body movements (70), and thus future rigorous studies are required to explore an association between emotional/behavioral problems and MPA in children with ASD.

## Conclusion

Results of this ASD children-based study found that the emotional symptoms were inversely associated with VPA and MVPA on the basis of our findings that no associations existed between PA and ASD-related deficiencies. Although the subgroup of mild-to-moderate ASD spent less daily VPA time than severe ASD in our study, the negative associations between emotional symptoms and VPA remained in mild-to-moderate and severe ASD, respectively. This means that the negative association between emotional symptoms and VPA was not altered by the difference in daily VPA duration between subgroups of mild-to-moderate and severe ASD. These results emphasize the importance of VPA and MVPA in children with ASD. Our findings justify the need to conduct the longitudinal and interventional research on the directionality of these associations. Also, further research is needed to examine the dose response required to have a benefit for emotional symptoms and to investigate which various emotional symptoms may be differentially affected by PA. Our findings offer potentially fruitful avenue for ASD children and their parents who are interested in improving emotional symptoms. If causal, these associations may also provide positive support to strategies aimed at alleviating emotional symptoms in individuals with ASD and may provide policy makers with an extra motivation to implement PA program with varying intensities.

## Limitations

In our study, the cross-sectional design nature and lack of a control group did not permit an investigation on causal association of objectively measured PA with mental, emotional and behavioral health. Future research is encouraged to make a more in-depth exploration of causality and mechanisms between emotional symptoms and SB, PA and step counts among ASD individuals. In addition, unequal gender distributions with smaller sample size of ASD girls were observed in our study due to the higher prevalence of ASD in boys. We will pay more attention to girls with ASD to compensate for unequal gender distributions in the future study.

## Data availability statement

The raw data supporting the conclusions of this article will be made available by the authors, without undue reservation.

## Ethics statement

Written informed consent was obtained from the individual(s), and minor(s)' legal guardian/next of kin, for the publication of any potentially identifiable images or data included in this article.

## Author contributions

JJ conceived the study and responsible for project administration. HL interpreted the data, conducted an in-depth analysis, and wrote the manuscript. YZ, BS, XW, MC, JC, SL, XZ, CJ, and ZG were responsible for data gathering. All authors contributed to the article and approved the submitted version.

## Funding

The study was funded by the Key-Area Research and Development Program of Guangdong Province (2019B030335001) and the National Natural Science Foundation of China (81872639).

## Conflict of interest

The authors declare that the research was conducted in the absence of any commercial or financial relationships that could be construed as a potential conflict of interest.

## Publisher's note

All claims expressed in this article are solely those of the authors and do not necessarily represent those of their affiliated organizations, or those of the publisher, the editors and the reviewers. Any product that may be evaluated in this article, or claim that may be made by its manufacturer, is not guaranteed or endorsed by the publisher.
