# Supplementary material for: Associations of emotional/behavioral problems with accelerometer-measured sedentary behavior, physical activity and step counts in children with autism spectrum disorder
Source: Front Public Health. 2022 Oct 10;10:981128. doi: 10.3389/fpubh.2022.981128 (PMC9588958; doi:10.3389/fpubh.2022.981128)
Supplement: Supplementary file 1 [file Table_1.docx]

**Table 1.** Comparisons of SDQ and SCARED scores between subgroups of intervention training, subgroups of accelerometer wear season in children with ASD.

| **Variables** | **Intervention training (n = 28)** | **No intervention training (n = 20)** | **Measuring in spring (n = 6)** | **Measuring in summer (n = 42)** |
| --- | --- | --- | --- | --- |
| SDQ |  | | | |
| Emotional symptoms | 2.00 ± 3.00 | 1.50 ± 4.00 | 2.50 ± 1.00 | 2.00 ± 4.00 |
| Conduct problems | 2.00 ± 3.00 | 2.00 ± 1.00 | 2.50 ± 2.00 | 2.00 ± 2.00 |
| Hyperactivity-inattention | 7.00 ± 4.00 | 7.50 ± 5.00 | 7.00 ± 5.00 | 7.00 ± 4.00 |
| Peer problems | 6.00 ± 3.00 | 6.00 ± 2.00 | 5.00 ± 3.00 | 6.00 ± 2.00 |
| Prosocial behavior | 5.00 ± 4.00 | 5.00 ± 3.00 | 4.00 ± 4.00 | 5.00 ± 3.00 |
| Total difficulties | 24.00 ± 9.00 | 21.00 ± 5.00 | 21.00 ± 7.00 | 23.00 ± 7.00 |
|  | **Intervention training (n = 30)** | **No intervention training (n = 16)** | **Measuring in spring (n = 20)** | **Measuring in summer (n = 26)** |
| SCARED |  | | | |
| Social phobia | 2.20 ± 1.10 | 1.75 ± 1.02 | 1.90 ± 1.42 | 2.10 ± 1.10 |
| Separation anxiety | 1.29 ± 0.46 | 1.14 ± 0.25 | 1.14 ± 0.25 | 1.29 ± 0.46 |
| Somatic/panic | 1.00 ± 0.20 | 1.10 ± 0.35 | 1.20 ± 0.35 | 1.00 ± 0.20 |
| Generalized anxiety | 2.00 ± 1.13 | 1.38 ± 0.94 | 1.75 ± 1.38 | 1.75 ± 1.06 |
| Total scores | 1.69 ± 0.59 | 1.48 ± 0.31 | 1.56 ± 0.49 | 1.67 ± 0.59 |

SDQ, strengths and difficulties questionnaire; SCARED, screen for child anxiety related emotional disorders; ASD, autism spectrum disorder; IQR, interquartile range.

Data were reported as median ± IQR. The Mann-Whitney U test was used to compare the SDQ and SCARED scores between different subgroups.

No statistically significant differences between subgroups of intervention training, subgroups of accelerometer wear season.

**Table 2.** Associations between accelerometer-measured SB, PA and step counts and RRBs (RBS-R) in children with ASD (n = 78).

| **Variables** | **β coefficient (95% confidence interval)** | | | | |  | **Odds ratio (95% confidence interval)** |
| --- | --- | --- | --- | --- | --- | --- | --- |
|  | **SB (hours/d)** | **LPA (min/d)** | **MPA (min/d)** | **VPA (min/d)** | **MVPA (min/d)** |  | **MVPA<60 min/d^a^** |
| Stereotypic behavior | –0.045 (–0.112, 0.021) | 2.129 (–1.338, 5.596) | 0.282 (–0.553, 1.117) | 0.311 (–0.381, 1.003) | 0.593 (–0.808, 1.995) |  | 0.936 (0.798, 1.099) |
| SIB | –0.081 (–0.188, 0.027) | 2.978 (–2.639, 8.594) | 0.997 (–0.339, 2.332) | 0.869 (–0.238, 1.976) | 1.866 (–0.372, 4.103) |  | 0.876 (0.677, 1.133) |
| Compulsive behavior | 0.006 (–0.064, 0.076) | –0.353 (–3.996, 3.290) | –0.136 (–1.007, 0.736) | 0.111 (–0.613, 0.834) | –0.025 (–1.491, 1.441) |  | 0.954 (0.807, 1.127) |
| Ritualistic behavior | –0.019 (–0.096, 0.059) | 1.186 (–2.827, 5.200) | 0.145 (–0.817, 1.107) | –0.211 (–1.008, 0.587) | –0.066 (–1.684, 1.552) |  | 0.958 (0.799, 1.149) |
| Sameness behavior | –0.008 (–0.053, 0.037) | 0.978 (–1.350, 3.305) | –0.220 (–0.777, 0.338) | –0.290 (–0.750, 0.170) | –0.509 (–1.443, 0.424) |  | 1.035 (0.915, 1.171) |
| Restricted interests | –0.055 (–0.129, 0.019) | 3.498 (–0.334, 7.330) | –0.065 (–1.000, 0.871) | –0.145 (–0.921, 0.631) | –0.210 (–1.782, 1.362) |  | 0.977 (0.821, 1.163) |
| Total scores | –0.007 (–0.022, 0.008) | 0.438 (–0.335, 1.211) | 0.005 (–0.181, 0.192) | –0.009 (–0.164, 0.146) | –0.004 (–0.317, 0.310) |  | 0.993 (0.958, 1.028) |
|  | **β coefficient (95% confidence interval)** | | | | | | |
|  | **Total PA (min/d)** | | **Thousand steps/d** | | **Steps/min** | | |
| Stereotypic behavior | 2.722 (–1.271, 6.715) | | 0.126 (–0.020, 0.272) | | 0.173 (–0.021, 0.368) | | |
| SIB | 4.843 (–1.595, 11.282) | | 0.208 (–0.028, 0.444) | | 0.285 (–0.030, 0.599) | | |
| Compulsive behavior | –0.378 (–4.583, 3.827) | | –0.079 (–0.233, 0.075) | | –0.099 (–0.304, 0.107) | | |
| Ritualistic behavior | 1.120 (–3.515, 5.756) | | –0.064 (–-0.234, 0.107) | | –0.070 (–0.298, 0.157) | | |
| Sameness behavior | 0.468 (–2.228, 3.165) | | –0.035 (–0.134, 0.064) | | –0.041 (–0.173, 0.091) | | |
| Restricted interests | 3.288 (–1.165, 7.741) | | 0.006 (–0.160, 0.172) | | 0.003 (–0.219, 0.224) | | |
| Total scores | 0.434 (–0.460, 1.328) | | 0.001 (–0.033, 0.034) | | 0.002 (–0.042, 0.047) | | |

RRBs, restricted repetitive behaviors; RBS-R, Repetitive Behavior Scale-Revised; ASD, autism spectrum disorder; SB, sedentary behavior; PA, physical activity; LPA, low PA;

MPA, moderate PA; VPA, vigorous PA; MVPA, moderate-to-vigorous PA; SIB, self-injurious behavior.

Adjusted for age, gender, severity of ASD symptoms, intellectual functioning, daily accelerometer wear time, maternal age, maternal educational level and monthly per-capita income.

^a^ Compared to MVPA≥60 min/d.

No significant associations between accelerometer-measured SB, PA and step counts and RRBs.

**Table 3.** Associations between accelerometer-measured SB, PA and step counts and social competence (SRS-2) in children with ASD (n = 78).

| **Variables** | **β coefficient (95% confidence interval)** | | | | | |  | **Odds ratio (95% confidence interval)** |
| --- | --- | --- | --- | --- | --- | --- | --- | --- |
|  | **SB (hours/d)** | **LPA (min/d)** | **MPA (min/d)** | **VPA (min/d)** | **MVPA (min/d)** | |  | **MVPA<60 min/d^a^** |
| Social awareness | –0.013 (–0.113, 0.086) | 0.482 (–4.687, 5.651) | –0.236 (–1.472, 1.000) | 0.558 (–0.461, 1.578) | 0.322 (–1.757, 2.401) | |  | 0.972 (0.769, 1.229) |
| Social cognition | –0.039 (–0.091, 0.013) | 2.314 (–0.366, 4.995) | –0.094 (–0.747, 0.559) | 0.120 (–0.422, 0.661) | 0.025 (–1.073, 1.123) | |  | 0.964 (0.851, 1.091) |
| Social communication | –0.003 (–0.034, 0.029) | 0.087 (–1.552, 1.727) | 0.003 (–0.389, 0.396) | 0.077 (–0.249, 0.402) | 0.080 (–0.579, 0.740) | |  | 0.955 (0.884, 1.031) |
| Social motivation | 0.002 (–0.049, 0.053) | 0.271 (–2.378, 2.920) | –0.141 (–0.774, 0.493) | –0.262 (–0.785, 0.261) | –0.403 (–1.465, 0.659) | |  | 0.996 (0.877, 1.130) |
| Autistic mannerisms | –0.009 (–0.048, 0.031) | 0.371 (–1.702, 2.444) | 0.033 (–0.463, 0.530) | 0.114 (–0.297, 0.525) | 0.147 (–0.687, 0.981) | |  | 0.917 (0.825, 1.019) |
| Total scores | –0.003 (–0.013, 0.008) | 0.151 (–0.405, 0.707) | –0.010 (–0.143, 0.123) | 0.017 (–0.094, 0.128) | 0.007 (–0.217, 0.231) | |  | 0.987 (0.961, 1.013) |
|  | **β coefficient (95% confidence interval)** | | | | | | | |
|  | **Total PA (min/d)** | | **Thousand steps/d** | | | **Steps/min** | | |
| Social awareness | 0.804 (–5.161, 6.769) | | –0.058 (–0.278, 0.161) | | | –0.079 (–0.371, 0.214) | | |
| Social cognition | 2.340 (–0.768, 5.447) | | 0.052 (–0.063, 0.168) | | | 0.065 (–0.089, 0.219) | | |
| Social communication | 0.167 (–1.725, 2.059) | | –0.012 (–0.081, 0.058) | | | –0.015 (–0.108, 0.078) | | |
| Social motivation | –0.132 (–3.190, 2.926) | | –0.017 (–0.129, 0.096) | | | –0.021 (–0.171, 0.129) | | |
| Autistic mannerisms | 0.518 (–1.873, 2.910) | | –0.014 (–0.102, 0.074) | | | –0.019 (–0.137, 0.098) | | |
| Total scores | 0.158 (–0.484, 0.800) | | –0.002 (–0.025, 0.022) | | | –0.002 (–0.034, 0.029) | | |

SRS-2, Social Responsiveness Scale Second Edition; ASD, autism spectrum disorder; SB, sedentary behavior; PA, physical activity; LPA, low PA; MPA, moderate PA; VPA,

vigorous PA; MVPA, moderate-to-vigorous PA.

Adjusted for age, gender, severity of ASD symptoms, intellectual functioning, daily accelerometer wear time, maternal age, maternal educational level and monthly per-capita income.

^a^ Compared to MVPA≥60 min/d.

No significant associations between accelerometer-measured SB, PA and step counts and social competence.

**Table 4.** Associations between accelerometer-measured SB, PA and step counts and motor development (DCDQ) in children with ASD (n = 78).

| **Variables** | **β coefficient (95% confidence interval)** | | | | |  | **Odds ratio (95% confidence interval)** |
| --- | --- | --- | --- | --- | --- | --- | --- |
|  | **SB (hours/d)** | **LPA (min/d)** | **MPA (min/d)** | **VPA (min/d)** | **MVPA (min/d)** |  | **MVPA<60 min/d^a^** |
| DCDQ total scores | 0.008 (–0.015, 0.032) | –0.345 (–1.575, 0.886) | –0.086 (–0.381, 0.208) | –0.064 (–0.308, 0.181) | –0.150 (–0.645, 0.345) |  | 1.560 (0.756, 3.221) |
| DCDQ result |  | | | | | | |
| DCD | –0.257 (–0.847, 0.333) | 7.142 (–23.630, 37.914) | 4.911 (–2.468, 12.290) | 3.371 (–2.819, 9.561) | 8.282 (–4.171, 20.735) |  | 0.370 (0.086, 1.594) |
| Suspect DCD | 0.331 (–0.215, 0.877) | –19.442 (–47.930, 9.046) | –1.299 (–8.131, 5.532) | 0.879 (–4.851, 6.610) | –0.420 (–11.949, 11.108) |  | 1.393 (0.353, 5.493) |
|  | **β coefficient (95% confidence interval)** | | | | | | |
|  | **Total PA (min/d)** | | **Thousand steps/d** | | **Steps/min** | | |
| DCDQ total scores | –0.495 (–1.913, 0.924) | | –0.017 (–0.069, 0.035) | | –0.024 (–0.094, 0.045) | | |
| DCDQ result |  | | | | | | |
| DCD | 15.424 (–19.953, 50.801) | | 0.558 (–0.770, 1.885) | | 0.752 (–1.019, 2.522) | | |
| Suspect DCD | –19.863 (–52.614, 12.889) | | 0.088 (–1.140, 1.317) | | 0.040 (–1.599, 1.679) | | |

DCDQ, developmental coordination disorder questionnaire; ASD, autism spectrum disorder; SB, sedentary behavior; PA, physical activity; LPA, low PA; MPA, moderate PA;

VPA, vigorous PA; MVPA, moderate-to-vigorous PA.

Adjusted for age, gender, severity of ASD symptoms, intellectual functioning, daily accelerometer wear time, maternal age, maternal educational level and monthly per-capita income.

^a^ Compared to MVPA≥60 min/d.

No significant associations between accelerometer-measured SB, PA and step counts and motor development.

**Table 5.** Associations of anxiety symptoms (SCARED) with accelerometer-measured SB, PA, and step counts in children with ASD (n = 46).

| **Variables** | **Odds ratio (95% confidence interval)** | | | | |
| --- | --- | --- | --- | --- | --- |
|  | **Social phobia** | **Separation anxiety** | **Somatic/panic** | **Generalized anxiety** | **Total scores** |
| SB (hours/d) |  |  |  |  |  |
| Crude model | 1.610 (0.975, 2.658) | 0.849 (0.509, 1.416) | 1.394 (0.819, 2.371) | 1.511 (0.929, 2.458) | 1.361 (0.852, 2.174) |
| Adjusted model | 1.439 (0.677, 3.059) | 0.314 (0.068, 1.449) | 0.842 (0.253, 2.798) | 1.015 (0.453, 2.273) | 1.259 (0.628, 2.524) |
| LPA (min/d) |  |  |  |  |  |
| Crude model | 0.998 (0.986, 1.009) | 1.005 (0.992, 1.017) | 1.004 (0.990, 1.018) | 0.997 (0.986, 1.009) | 0.997 (0.986, 1.009) |
| Adjusted model | 0.995 (0.980, 1.009) | 1.020 (0.993, 1.047) | 1.007 (0.982, 1.032) | 1.004 (0.988, 1.020) | 0.997 (0.984, 1.011) |
| MPA (min/d) |  |  |  |  |  |
| Crude model | 0.985 (0.943, 1.029) | 0.989 (0.942, 1.039) | 0.998 (0.947, 1.051) | 0.974 (0.930, 1.019) | 0.981 (0.938, 1.025) |
| Adjusted model | 0.959 (0.899, 1.024) | 1.013 (0.924, 1.110) | 1.005 (0.922, 1.095) | 0.963 (0.895, 1.037) | 0.975 (0.920, 1.033) |
| VPA (min/d) |  |  |  |  |  |
| Crude model | 0.999 (0.945, 1.055) | 1.035 (0.975, 1.098) | 0.993 (0.927, 1.062) | 0.928 (0.866, 0.994)^*^ | 0.976 (0.922, 1.033) |
| Adjusted model | 0.986 (0.912, 1.065) | 1.041 (0.950, 1.141) | 0.945 (0.838, 1.066) | 0.900 (0.798, 1.015) | 0.970 (0.902, 1.043) |
| MVPA (min/d) |  |  |  |  |  |
| Crude model | 0.994 (0.968, 1.021) | 1.004 (0.975, 1.034) | 0.997 (0.965, 1.031) | 0.973 (0.945,1.003) | 0.987 (0.960, 1.014) |
| Adjusted model | 0.982 (0.945, 1.020) | 1.016 (0.967, 1.068) | 0.991 (0.941, 1.043) | 0.964 (0.917, 1.013) | 0.984 (0.950, 1.019) |
| MVPA≥60 min/d^a^ |  |  |  |  |  |
| Crude model | 0.778 (0.230, 2.634) | 1.250 (0.330, 4.732) | 1.333 (0.315, 5.642) | 0.398 (0.111, 1.427) | 0.778 (0.230, 2.634) |
| Adjusted model | 0.505 (0.103, 2.482) | 2.265 (0.290, 17.698) | 0.723 (0.075, 6.920) | 0.203 (0.022, 1.881) | 0.675 (0.146, 3.118) |
| Total PA (min/d) |  |  |  |  |  |
| Crude model | 0.997 (0.988, 1.007) | 1.004 (0.993, 1.015) | 1.003 (0.991, 1.014) | 0.995 (0.985, 1.005) | 0.996 (0.987, 1.006) |
| Adjusted model | 0.994 (0.982, 1.007) | 1.019 (0.994, 1.046) | 1.003 (0.983, 1.023) | 1.000 (0.986, 1.013) | 0.996 (0.985, 1.008) |
| Thousand steps/d |  |  |  |  |  |
| Crude model | 0.881 (0.689, 1.127) | 1.065 (0.817, 1.390) | 1.016 (0.761, 1.356) | 0.839 (0.650, 1.081) | 0.893 (0.699, 1.141) |
| Adjusted model | 0.865 (0.617, 1.214) | 1.160 (0.732, 1.837) | 1.160 (0.698, 1.928) | 0.902 (0.583, 1.397) | 0.909 (0.665, 1.242) |
| Steps/min |  |  |  |  |  |
| Crude model | 0.869 (0.723, 1.044) | 1.057 (0.877, 1.275) | 0.953 (0.773, 1.175) | 0.864 (0.718, 1.041) | 0.903 (0.756, 1.078) |
| Adjusted model | 0.888 (0.688, 1.145) | 1.124 (0.793, 1.595) | 1.121 (0.762, 1.649) | 0.942 (0.682, 1.302) | 0.926 (0.733, 1.170) |

SCARED, screen for child anxiety related emotional disorders; ASD, autism spectrum disorder; SB, sedentary behavior; PA, physical activity; LPA, low PA;

MPA, moderate PA; VPA, vigorous PA; MVPA, moderate-to-vigorous PA. Crude model: no adjust. Adjusted model: adjusted for age, gender, severity of ASD symptoms, intellectual functioning, daily accelerometer wear time, maternal age, maternal educational level and monthly per-capita income. ^a^ Compared to MVPA<60 min/d.

^*^ Statistically significant associations (*p* < 0.05).

**Table 6.** Associations of the levels of emotional/behavioral problems (SDQ) with IPAQ-SF assessed SB, PA and walking in children with ASD (n = 48).

| **Variables** | **β coefficient (95% confidence interval)** | | | | | |
| --- | --- | --- | --- | --- | --- | --- |
|  | **Emotional symptoms** | **Conduct problems** | **Hyperactivity-inattention** | **Peer problems** | **Prosocial behavior** | **Total difficulties** |
| SB (hours/d) |  |  |  |  |  |  |
| Crude model | 0.272 (0.075, 0.468)^**^ | 0.074 (–0.074, 0.221) | 0.095 (–0.161, 0.351) | –0.050 (–0.251, 0.150) | 0.239 (0.003, 0.474)^*^ | 0.390 (–0.119, 0.899) |
| Adjusted model | 0.251 (0.034, 0.467)^*^ | –0.003 (–0.147, 0.142) | 0.130 (–0.144, 0.404) | 0.021 (–0.181, 0.223) | 0.142 (–0.076, 0.360) | 0.399 (–0.182, 0.980) |
| MPA (min/d) |  |  |  |  |  |  |
| Crude model | –0.010 (–0.021, 0.001) | –0.005 (–0.013, 0.003) | 0.000 (–0.014, 0.014) | 0.004 (–0.007, 0.014) | –0.009 (–0.022, 0.003) | –0.011 (–0.039, 0.016) |
| Adjusted model | –0.012 (–0.023, –0.001)^*^ | 0.000 (–0.008, 0.007) | –0.001 (–0.015, 0.013) | –0.004 (–0.015, 0.006) | –0.006 (–0.017, 0.005) | –0.018 (–0.048, 0.011) |
| VPA (min/d) |  |  |  |  |  |  |
| Crude model | –0.018 (–0.031, –0.004)^*^ | –0.003 (–0.013, 0.007) | 0.012 (–0.005, 0.029) | –0.008 (–0.014, 0.014) | –0.012 (–0.028, 0.005) | –0.008 (–0.043, 0.027) |
| Adjusted model | –0.020 (–0.033, –0.006)^**^ | 0.002 (–0.007, 0.011) | 0.008 (–0.010, 0.025) | –0.008 (–0.021, 0.004) | –0.003 (–0.017, 0.012) | –0.018 (–0.056, 0.019) |
| MVPA (min/d) |  |  |  |  |  |  |
| Crude model | –0.009 (–0.017, –0.002)^**^ | –0.003 (–0.008, 0.002) | 0.003 (–0.006, 0.013) | 0.002 (–0.006, 0.009) | –0.007 (–0.016, 0.001) | –0.007 (–0.026, 0.011) |
| Adjusted model | –0.011 (–0.018, –0.004)^**^ | 0.000 (–0.004, 0.005) | 0.001 (–0.008, 0.011) | –0.004 (–0.011, 0.002) | –0.003 (–0.011, 0.004) | –0.013 (–0.033, 0.006) |
| Walking (min/d) |  |  |  |  |  |  |
| Crude model | –0.003 (–0.017, 0.010) | –0.003 (–0.013, 0.006) | –0.009 (–0.025, 0.007) | 0.004 (–0.009, 0.016) | 0.002 (–0.013, 0.018) | –0.012 (–0.045, 0.021) |
| Adjusted model | –0.005 (–0.018, 0.009) | –0.004 (–0.013, 0.004) | –0.004 (–0.021, 0.012) | 0.002 (–0.010, 0.014) | –0.003 (–0.016, 0.010) | –0.011 (–0.046, 0.024) |

SDQ, Strengths and difficulties questionnaire; IPAQ-SF, International Physical Activity Questionnaire-Short Form; SB, sedentary behavior; PA, physical activity;

MPA, moderate PA; VPA, vigorous PA; MVPA, moderate-to-vigorous PA; ASD, autism spectrum disorder. .

Adjusted model: adjusted for age, gender, severity of ASD symptoms, intellectual functioning, daily accelerometer wear time, maternal age,

maternal educational level and monthly per-capita income.

^*^ Statistically significant associations (*p* < 0.05); ^**^ Statistically significant associations (*p* < 0.01).

**Table 7.** Associations of emotional/behavioral problems (SDQ) with IPAQ-SF assessed SB, PA and walking in children with ASD (n = 48).

| **Variables** | **Odds ratio (95% confidence interval)** | | | | | |
| --- | --- | --- | --- | --- | --- | --- |
|  | **Emotional symptoms** | **Conduct problems** | **Hyperactivity-inattention** | **Peer problems** | **Prosocial behavior** | **Total difficulties** |
| SB (hours/d) |  |  |  |  |  |  |
| Crude model | 1.289 (1.010, 1.644)^*^ | 1.162 (0.925, 1.459) | 1.119 (0.855, 1.465) | 0.917 (0.637, 1.320) | 0.863 (0.681, 1.094) | 1.205 (0.867, 1.673) |
| Adjusted model | 1.427 (0.998, 2.041) | 0.980 (0.697, 1.378) | 1.240 (0.813, 1.890) | 1.120 (0.535, 2.343) | 0.780 (0.541, 1.125) | 1.236 (0.786, 1.942) |
| MPA (min/d) |  |  |  |  |  |  |
| Crude model | 0.992 (0.977, 1.007) | 0.995 (0.982, 1.009) | 0.999 (0.986, 1.012) | 1.010 (0.981, 1.040) | 1.012 (0.994, 1.030) | 0.990 (0.977, 1.004) |
| Adjusted model | 0.982 (0.956, 1.008) | 1.011 (0.990, 1.032) | 1.000 (0.983, 1.017) | 1.026 (0.969, 1.087) | 1.033 (1.001, 1.067) | 0.982 (0.964, 1.001) |
| VPA (min/d) |  |  |  |  |  |  |
| Crude model | 0.981 (0.960, 1.003) | 0.997 (0.981, 1.013) | 1.006 (0.988, 1.025) | 0.987 (0.966, 1.009) | 1.020 (0.997, 1.044) | 0.999 (0.981, 1.017) |
| Adjusted model | 0.966 (0.935, 0.998)^*^ | 1.008 (0.982, 1.036) | 1.002 (0.976, 1.029) | 0.158 (0.000, 1.551) | 1.054 (1.006, 1.105) | 0.996 (0.971, 1.023) |
| MVPA (min/d) |  |  |  |  |  |  |
| Crude model | 0.990 (0.978, 1.002) | 0.997 (0.988, 1.006) | 1.001 (0.992, 1.011) | 0.999 (0.986, 1.012) | 1.001 (0.985, 1.016) | 0.995 (0.987, 1.004) |
| Adjusted model | 0.979 (0.958, 0.999)^*^ | 1.007 (0.993, 1.021) | 1.000 (0.988, 1.013) | 0.990 (0.954, 1.027) | 1.037 (1.004, 1.072) | 0.990 (0.978, 1.003) |
| Walking (min/d) |  |  |  |  |  |  |
| Crude model | 1.001 (0.986, 1.015) | 1.000 (0.985, 1.014) | 0.991 (0.976, 1.006) | 0.994 (0.974, 1.014) | 1.205 (0.867, 1.673) | 0.988 (0.972, 1.004) |
| Adjusted model | 1.003 (0.986, 1.022) | 1.005 (0.982, 1.029) | 0.997 (0.977, 1.018) | 0.994 (0.950, 1.040) | 1.013 (0.992, 1.035) | 0.986 (0.963, 1.009) |

SDQ, Strengths and difficulties questionnaire; IPAQ-SF, International Physical Activity Questionnaire-Short Form; SB, sedentary behavior; PA, physical activity;

MPA, moderate PA; VPA, vigorous PA; MVPA, moderate-to-vigorous PA; ASD, autism spectrum disorder. .

Adjusted model: adjusted for age, gender, severity of ASD symptoms, intellectual functioning, daily accelerometer wear time, maternal age,

maternal educational level and monthly per-capita income.

^*^ Statistically significant associations (*p* < 0.05).

**Table 8.** Associations of anxiety levels (SCARED) with IPAQ-SF assessed SB, PA and walking in children with ASD (n = 46).

| **Variables** | **β coefficient (95% confidence interval)** | | | | |
| --- | --- | --- | --- | --- | --- |
|  | **Social phobia** | **Separation anxiety** | **Somatic/panic** | **Generalized anxiety** | **Total scores** |
| SB (hours/d) |  |  |  |  |  |
| Crude model | 0.041 (–0.031, 0.114) | 0.015 (–0.023, 0.054) | 0.021 (–0.005, 0.048) | 0.055 (–0.025, 0.135) | 0.033 (–0.009, 0.076) |
| Adjusted model | 0.012 (–0.072, 0.096) | 0.021 (–0.025, 0.067) | 0.001 (–0.029, 0.030) | 0.016 (–0.077, 0.110) | 0.014 (–0.036, 0.064) |
| MPA (min/d) |  |  |  |  |  |
| Crude model | –0.001 (–0.005, 0.003) | 0.000 (–0.002, 0.002) | 0.000 (–0.002, 0.001) | 0.002 (–0.003, 0.006) | 0.001 (–0.003, 0.002) |
| Adjusted model | 0.000 (–0.004, 0.005) | 0.001 (–0.001, 0.004) | 0.000 (–0.002, 0.001) | 0.003 (–0.001, 0.008) | 0.001 (–0.002, 0.003) |
| VPA (min/d) |  |  |  |  |  |
| Crude model | –0.005 (–0.010, 0.000) | –0.001 (–0.004, 0.001) | –0.001 (–0.003, 0.001) | –0.003 (–0.009, 0.002) | –0.003 (–0.006, 0.000) |
| Adjusted model | –0.006 (–0.011, –0.001)^*^ | –0.001 (–0.004, 0.002) | –0.002 (–0.003, 0.000) | –0.004 (–0.009, 0.002) | –0.003 (–0.006, –0.001)^*^ |
| MVPA (min/d) |  |  |  |  |  |
| Crude model | –0.002 (–0.004, 0.001) | 0.000 (–0.002, 0.001) | 0.000 (–0.001, 0.000) | 0.000 (–0.003, 0.003) | –0.001 (–0.002, 0.001) |
| Adjusted model | –0.001 (–0.004, 0.001) | 0.000 (–0.001, 0.002) | –0.001 (–0.002, 0.000) | 0.000 (–0.003, 0.003) | –0.001 (–0.002, 0.001) |
| Walking (min/d) |  |  |  |  |  |
| Crude model | 0.001 (–0.003, 0.005) | –0.001 (–0.004, 0.001) | 0.001 (–0.002, 0.002) | 0.005 (0.000, 0.009)^*^ | 0.001 (–0.002, 0.003) |
| Adjusted model | 0.003 (–0.001, 0.008) | –0.001 (–0.003, 0.002) | 0.001 (–0.001, 0.002) | 0.006 (0.002, 0.011) | 0.002 (0.000, 0.005) |

SCARED, screen for child anxiety related emotional disorders; IPAQ-SF, International Physical Activity Questionnaire-Short Form; SB, sedentary behavior; PA, physical

Activity; MPA, moderate PA; VPA, vigorous PA; MVPA, moderate-to-vigorous PA; ASD, autism spectrum disorder. .

Adjusted model: adjusted for age, gender, severity of ASD symptoms, intellectual functioning, daily accelerometer wear time, maternal age,

maternal educational level and monthly per-capita income.

^*^ Statistically significant associations (*p* < 0.05).

**Table 9.** Associations of anxiety symptoms (SCARED) with IPAQ-SF assessed SB, PA and walking in children with ASD (n = 46).

| **Variables** | **Odds ratio (95% confidence interval)** | | | | |
| --- | --- | --- | --- | --- | --- |
|  | **Social phobia** | **Separation anxiety** | **Somatic/panic** | **Generalized anxiety** | **Total scores** |
| SB (hours/d) |  |  |  |  |  |
| Crude model | 1.132 (0.904, 1.417) | 0.986 (0.772, 1.260) | 1.117 (0.865, 1.442) | 1.243 (0.981, 1.575) | 1.168 (0.929, 1.468) |
| Adjusted model | 1.044 (0.751, 1.452) | 0.937 (0.620, 1.416) | 0.710 (0.357, 1.411) | 1.069 (0.679, 1.683) | 1.047 (0.761, 1.442) |
| MPA (min/d) |  |  |  |  |  |
| Crude model | 0.998 (0.985, 1.011) | 1.003 (0.990, 1.017) | 0.996 (0.979, 1.013) | 1.000 (0.987, 1.012) | 0.997 (0.984, 1.010) |
| Adjusted model | 1.002 (0.986, 1.018) | 1.011 (0.993, 1.031) | 0.986 (0.961, 1.011) | 1.010 (0.989, 1.031) | 0.999 (0.984, 1.014) |
| VPA (min/d) |  |  |  |  |  |
| Crude model | 0.988 (0.972, 1.006) | 0.995 (0.977, 1.013) | 0.986 (0.963, 1.010) | 0.993 (0.977, 1.009) | 0.993 (0.978, 1.009) |
| Adjusted model | 0.986 (0.967, 1.006) | 1.000 (0.979, 1.023) | 0.971 (0.940, 1.003) | 0.981 (0.955, 1.009) | 0.992 (0.974, 1.011) |
| MVPA (min/d) |  |  |  |  |  |
| Crude model | 0.996 (0.988, 1.005) | 1.000 (0.991, 1.009) | 0.994 (0.982, 1.007) | 0.998 (0.990, 1.006) | 0.997 (0.989, 1.005) |
| Adjusted model | 0.997 (0.986, 1.008) | 1.004 (0.993, 1.016) | 0.988 (0.972, 1.004) | 0.999 (0.986, 1.012) | 0.997 (0.987, 1.007) |
| Walking (min/d) |  |  |  |  |  |
| Crude model | 1.001 (0.989, 1.014) | 0.999 (0.985, 1.013) | 0.995 (0.978, 1.013) | 1.013 (0.997, 1.029) | 1.006 (0.993, 1.020) |
| Adjusted model | 1.008 (0.991, 1.025) | 1.001 (0.979, 1.023) | 1.004 (0.982, 1.026) | 1.030 (0.999, 1.062) | 1.016 (0.997, 1.036) |

SCARED, screen for child anxiety related emotional disorders; IPAQ-SF, International Physical Activity Questionnaire-Short Form; SB, sedentary behavior; PA, physical activity; MPA, moderate PA; VPA, vigorous PA; MVPA, moderate-to-vigorous PA; ASD, autism spectrum disorder. .

Adjusted model: adjusted for age, gender, severity of ASD symptoms, intellectual functioning, daily accelerometer wear time, maternal age,

maternal educational level and monthly per-capita income.

^*^ Statistically significant associations (*p* < 0.05).

**Table 10.** Associations of the levels of emotional symptoms (SDQ) with accelerometer-measured PA in mild-to-moderate and severe ASD children.

| **Variables** | **β coefficient (95% confidence interval)** | |
| --- | --- | --- |
|  | **Mild-to-moderate ASD (n=36)** | **Severe ASD (n=12)** |
| VPA (min/d) |  |  |
| Crude model | –0.082 (–0.152, –0.001)^*^ | –0.045 (–0.121, 0.030) |
| Adjusted model | –0.087 (–0.166, –0.009)^*^ | –0.129 (–0.165, –0.094)^**^ |
| MVPA (min/d) |  |  |
| Crude model | –0.023 (–0.061, 0.015) | –0.029 (–0.068, 0.010) |
| Adjusted model | –0.017 (–0.064, 0.030) | –0.074 (–0.084, –0.064)^**^ |

SDQ, strengths and difficulties questionnaire; ASD, autism spectrum disorder; PA, physical activity;

VPA, vigorous PA; MVPA, moderate-to-vigorous PA.

Crude model: no adjust. Adjusted model: adjusted for age, gender, intellectual functioning,

daily accelerometer wear time, maternal age and monthly per-capita income.

^*^ Statistically significant associations (*p* < 0.05); ^**^ Statistically significant associations (*p* < 0.01).
